# Magnetic Chitosan Bionanocomposite Films as a Versatile Platform for Biomedical Hyperthermia

**DOI:** 10.1002/adhm.202303861

**Published:** 2023-12-13

**Authors:** Ana Barra, Jacek K. Wychowaniec, Danielle Winning, Maria Margarida Cruz, Liliana P. Ferreira, Brian J. Rodriguez, Helena Oliveira, Eduardo Ruiz‐Hitzky, Cláudia Nunes, Dermot F. Brougham, Paula Ferreira

**Affiliations:** ^1^ Department of Materials and Ceramic Engineering CICECO – Aveiro Institute of Materials University of Aveiro Aveiro 3810—193 Portugal; ^2^ Materials Science Institute of Madrid CSIC c/Sor Juana Inés de la Cruz 3 Madrid 28049 Spain; ^3^ School of Chemistry University College Dublin Belfield Dublin D04 V1W8 Ireland; ^4^ AO Research Institute Davos Clavadelerstrasse 8 Davos 7270 Switzerland; ^5^ Biosystems and Integrative Sciences Institute (BioISI) Faculdade de Ciências Universidade de Lisboa Lisboa 1749‐016 Portugal; ^6^ Physics Department University of Coimbra Coimbra 3004—516 Portugal; ^7^ School of Physics and Conway Institute of Biomolecular and Biomedical Research University College Dublin Belfield Dublin D04 V1W8 Ireland; ^8^ Department of Biology and CESAM University of Aveiro Aveiro 3810‐193 Portugal

**Keywords:** bionanocomposite films, chitosan, hydrogels, magnetic hyperthermia, magnetite, melanoma cells

## Abstract

Responsive magnetic nanomaterials offer significant advantages for innovative therapies, for instance, in cancer treatments that exploit on‐demand delivery on alternating magnetic field (AMF) stimulus. In this work, biocompatible magnetic bionanocomposite films are fabricated from chitosan by film casting with incorporation of magnetite nanoparticles (MNPs) produced by facile one pot synthesis. The influence of synthesis conditions and MNP concentration on the films’ heating efficiency and heat dissipation are evaluated through spatio‐temporal mapping of the surface temperature changes by video‐thermography. The cast films have a thickness below 100 µm, and upon exposure to AMF (663 kHz, 12.8 kA m^−1^), induce exceptionally strong heating, reaching a maximum temperature increase of 82 °C within 270 s irradiation. Further, it is demonstrated that the films can serve as substrates that supply heat for multiple hyperthermia scenarios, including: i) non‐contact automated heating of cell culture medium, ii) heating of gelatine‐based hydrogels of different shapes, and iii) killing of cancerous melanoma cells. The films are versatile components for non‐contact stimulus with translational potential in multiple biomedical applications.

## Graphical abstract

The strong heating efficiency of magnetic chitosan bionanocomposite films, with thickness below 100 µm, is studied by video thermography under alternating magnetic field (AMF). High hyperthermia (MH) efficiency and precise temperature control using AMF are demonstrated by MH treatment of MNT‐1 cells. The cell's viability drastically decreases below 10%, 48 h after a single AMF cycle of 10 min.

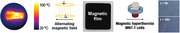

## 1 Introduction

Magnetic iron oxide nanoparticles (MNPs) and their clusters are used in a wide range of biomedical applications from cancer treatment to magnetic resonance imaging.^[^ ^1^, ^2^ ^]^ Responsive magnetic materials have been proposed as functional components for cancer therapies that may provide spatiotemporal‐controlled drug release actuated by magnetic hyperthermia (MH) under alternating magnetic field (AMF) stimulation.^[^ ^3^, ^4^, ^5^ ^]^ The approach relies on heat dissipation by MNPs under AMF, which is feasible as magnetic fields permeate most soft hydrated materials including the human body.^[^ ^6^, ^7^ ^]^ MNPs are widely used for MH cancer therapy due to their high magnetization, optimizable moment dynamics, biocompatibility, biodegradability, and relatively easy synthesis.^[^ ^8^, ^9^ ^]^ Such approaches require stable colloidal MNP suspensions which are injected in the tumor area at sufficient concentration so that, on exposure to AMF, the local temperature increases above physiological and often up to 41–45 °C.^[^ ^10^ ^]^ This triggers apoptosis mechanisms in tumor cells, which are usually temperature‐sensitive.^[^ ^11^, ^12^, ^13^, ^14^ ^]^ MNP‐based formulations are approved for treatment of tumors in humans by MH. In 2013, the NanoTherm formulation, composed of aminosilane coated iron oxide nanoparticles, was authorized for use in Europe. Clinical trials for its use in treatment of prostate carcinoma began in the US in 2018;^[^ ^15^ ^]^ while clinical trials for treatment of advanced pancreatic carcinoma, using a suspension of MNPs coated with dextran, re also currently ongoing in Spain.^[^ ^16^, ^17^ ^]^

Despite the current clinical status, MH cancer therapy has serious limitations. For instance, poor MNP dispersion (i.e., changes in homogeneity, colloidal stability; and hence, concentration both before and after administration) can affect heating efficiency and uniformity of distribution in the tumor, resulting in poor dose control and non‐uniform heating profiles. On the other hand rapid intracellular biodegradation of MNPs can result in insufficient heat dose in the target area, reducing treatment efficiency.^[^ ^18^ ^]^ Further, intravenously, and even intratumorally, administered nanoparticles can be found in healthy tissue.^[^ ^19^, ^20^ ^]^ Many surface coating strategies, including grafting with polyethyleneglycol and other molecules, have been developed to improve chemical and colloidal stability and particle retention.^[^ ^21^, ^22^, ^23^, ^24^, ^25^, ^26^, ^27^ ^]^

Dispersion and stabilization of MNPs in polymers to form responsive nanocomposites is an alternative approach that may overcome many limitations of suspension‐based MH. Nanocomposite materials which combine MNPs with chemically defined polymers or hydrogels, in bulk or film formats, provide field‐responsiveness^[^ ^3^, ^4^, ^28^, ^29^ ^]^ and particle retention/localization. In previous work, we have shown that MNPs can be stabilized in patternable synthetic hydrogels and that the MH thermo‐response of the nanocomposite is controlled by concentration‐dependent interactions between MNPs that are modulated by their surface chemistry.^[^ ^5^ ^]^ Natural biopolymers are attractive platforms for development of bionanocomposites due to their non‐toxicity, biocompatibility, and biodegradability.^[^ ^30^, ^31^ ^]^ For example, chitosan‐MNP nanocomposite hydrogels for drug delivery applications have been reported for which the particles also provide magnetophoretic positioning at the target location.^[^ ^32^ ^]^ MNPs have even been reported to improve biocompatibility of bionanocomposites, as indicated by increased cell viability rates when incorporated in alginate‐chitosan multilayer films.^[^ ^33^ ^]^

The use of magnetic bionanocomposites as external adhesive pads or surgically implantable materials for dose‐controlled localized MH therapy has been suggested. Chen et al.^[^ ^34^ ^]^ evaluated the hyperthermic efficacy of MNPs and MNP/alginate/polyvinyl alcohol mats, at the same MNP concentration, against A‐549 human lung carcinoma cells. The mats showed a 60% increased hyperthermic effect as compared to colloidal MNPs due to enhanced cell contact. However, the mats displayed a lower heating efficiency in comparison with MNPs, which was attributed to poor thermal conductivity of matrix. Criado et al.^[^ ^35^ ^]^ also used MNP‐alginate‐chitosan multilayer films for MH treatment of neuroblastoma cells. Viability of cells cultured on the top of the films was reduced to 69% and 21% with one or three AMF cycles (30 min on, with 5 h waiting time), respectively. In our previous work, MH response of multifunctional chitosan‐based films containing rGO‐Fe_3−_ *_x_* O_4−_ *_x_* prepared by solvent casting was evaluated.^[^ ^36^ ^]^ The dry film containing 50 wt% rGO‐Fe_3−_ *_x_* O_4−_ *_x_* showed a temperature increase, Δ*T*, of 40 °C in only 45 s under AMF. In addition, these films revealed non‐cytotoxicity toward the non‐tumorigenic human keratinocyte cells (HaCat cell line). To realize the potential of these materials for MH, it is necessary to study the relationship between composition and AMF‐induced thermal response. Moreover, it is necessary to ensure the film integrity upon contact with liquid medium retention of heating efficiency in complex media.^[^ ^34^ ^]^ The design of highly efficient MH materials, that allow precise temperature control in spatially defined volumes is a necessary step toward spatially localizable MH treatments with controlled heat dose. In addition, increased MH efficiency is highly desirable to reduce the duration and number of treatment cycles required to significantly reduce cancer cell viability.

In this study, chitosan‐based bionanocomposite films containing MNPs synthesized by co‐precipitation are prepared by a simple solvent casting method using non‐toxic materials. The effects of film thickness and MNP content on heating performance under AMF are investigated, identifying the synthetic conditions for preparing exceptionally high‐heating films. The MH performance of the film with the highest heating response is investigated in different biomedically relevant scenarios. The film is used to heat cell culture medium and gelatine‐based hydrogels of different shapes. The film is also used for the MH treatment of MNT‐1 human melanoma cells. Spatiotemporally‐resolved heat mapping of the bionanocomposite films under AMF (663 kHz, 12.8 kA m^−1^) is described for the first time, using an in situ combined thermal camera with a live‐cell alternating magnetic field (LC‐AMF) setup. Particle homogeneity across the films in both the lateral and vertical directions is evaluated, which underpins interpretation of heat flow and reveals the response of the films upper surface which is in direct contact with the cells or the medium.

## 2 Results and Discussion

### 2.1 Characterization of MNPs

**Figure 1:**
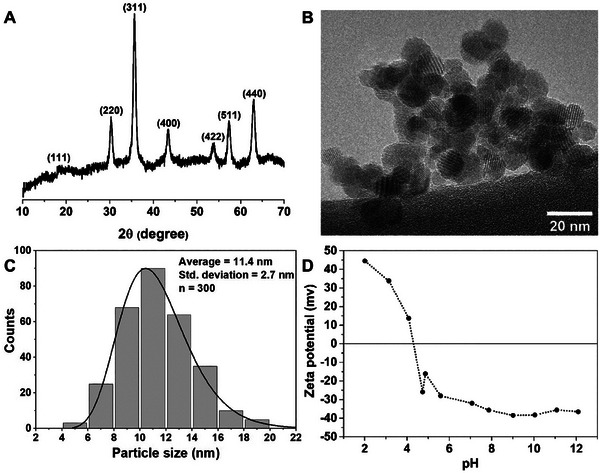
A) X‐ray diffraction pattern of magnetic iron‐oxide MNPs. B) A representative TEM image of MNPs. C) TEM particle size distribution histogram with a log‐normal fit. D) Zeta potential profile of an 0.07 mg mL^−1^ aqueous suspension of MNPs.

The MNPs, prepared by the co‐precipitation method, were black in appearance and the magnetite crystalline phase was confirmed by XRD, **Figure** **1**A. The peak positions at 2*θ* 18.6°, 30.2°, 35.6°, 43.2°, 53.8°, 57.4°, 63.0°, indexed to the Fe_3_O_4_ diffraction planes (111), (220), (311), (400), (422), (511), (440), respectively, were in good agreement with JCPDS card 19–0629.

The particle morphology, size, and distribution were studied by TEM. A representative image is shown in Figure 1B, which shows mostly crystalline almost spherical particles. The average particle diameter of 11.4 ± 2.7 nm, Figure 1C, is as expected for synthesis by co‐precipitation.^[^ ^37^ ^]^ Some MNP aggregates are observed in the images, which we attribute to aggregation during drying of the suspension onto the TEM grid. The zeta potential response of the suspension changes from positive to negative with increasing pH, with the isoelectric point identified at pH 4.3 ± 0.1, Figure 1D.

### 2.2 Topography, Elemental Composition, and Magnetic Properties of Bionanocomposite Films

**Figure 2:**
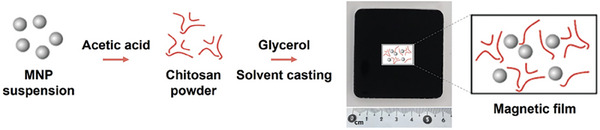
Schematic representation of magnetic bionanocomposite film preparation. The black square in the picture with the ruler is a representative photograph of a film sample.

Bionanocomposite films were prepared according to the scheme shown in **Figure** **2**. In brief, chitosan was dissolved in MNP suspensions using 0.1 m acetic acid, that is, in the acidic pH range of 2–3. At this pH, MNPs have high positive zeta potential values, in the range 35–45 mV, Figure 1D, indicative of stable fully dispersed individual MNPs in suspension.^[^ ^22^ ^]^ Chitosan has previously been shown to contribute to stabilization of MNPs preventing their aggregation.^[^ ^38^ ^]^ Two different concentrations of MNPs (1.5 w/v% and 2.25 w/v%) and glycerol (0.38 w/v% and 0.75 w/v%) were used for the preparation of the films, **Table** **1**. Film thickness was controlled, during the solvent casting, simply by adjusting the quantity of the solution in the acrylic casting mold, to give thin or thick films, Table 1. The different samples were named as; *x*M_*y*G_t/T where *x and y* stand for concentrations (w/v%) of magnetite (M) and glycerol (G), respectively, and with (t) denoting thin and (T) thick films.

**Table 1:** Nominal composition and thickness of the magnetic bionanocomposite films.

Sample	Chitosan [w/v%]	Fe_3_O_4_ [w/v%]	Glycerol [w/v%]	Thickness [µm]
1.5M_0.75G_t	1.5	1.5	0.75	37 ± 4^c^
1.5M_0.75G_T	1.5	1.5	0.75	93 ± 10^a^
1.5M_0.38G_t	1.5	1.5	0.38	34 ± 1^c^
2.25M_0.75G_t	1.5	2.25	0.75	42 ± 1^c^
2.25M_0.75G_T	1.5	2.25	0.75	78 ± 6^b^
2.25M_0.38G_t	1.5	2.25	0.38	35 ± 3^c^

Thickness means with different letters are statistically different, *p* value < 0.05.

The morphology and elemental composition of the films are investigated by SEM‐EDS. SEM images of the upper surface, Figure S1, Supporting Information reveal no major differences between the different samples, apart from a slight increase in particle number for the higher MNP concentration films and no significant porosity. There is no evidence of aggregates on the surface. Following co‐precipitation, aggregates of the scale of 100 nm are expected,^[^ ^39^ ^]^ clearly reducing the pH and subsequent addition of chitosan help to disperse the MNPs. SEM images of the lower surfaces are of poorer quality. We suggest that this is due to: i) higher glycerol content giving rise to more decomposition of organics under the electron beam and ii) deposition of MNPs (by gravity) during the solvent casting process. Higher MNP content closer to the bottom surface will affect the image quality, even in the magnetic field‐free detection mode used.

EDS analysis, **Table** **2**, confirms higher Fe content on the bottom side for all films, stemming from partial sedimentation prior to full solvent evaporation. The ratio of the Fe wt% top/bottom varies from 0.42 to 0.67, with no trend apparent. The Fe content is also determined by ICP‐OES after full digestion of the films with nitric acid, Table 2. The analysis confirms no loss of Fe from the initial formulations (average incorporation 108% ± 7%). In all cases, the average of the (top and bottom) EDS Fe concentrations are the same, within error, as those from ICP‐OES. This is consistent with a linear vertical gradient in Fe concentration for all compositions and supports the absence of significant Fe loss.

**Table 2:** Nominal concentration, (N.C.), of Fe_3_O_4_ (wt%) and corresponding Fe (wt%) added to the bionanocomposite film formulations. Fe (wt%) in the magnetic films determined by ICP‐OES and the Fe (%) incorporated. Relative Fe weight percentages (wt%) on the top and bottom of magnetic films determined by EDS and the top/bottom ratio. Saturation magnetisation, *M*_s_ (emu g^−1^ of film) values obtained by SQUID magnetometry. The temperature increase at the plateau, Δ*T*_max_ (°C), achieved by each film during AMF stimulation.

Sample	N. C. [wt%]		ICP‐OES	EDS ‐ Fe [wt%]			*M*_s_ [emu g^−1^]		Δ*T*_max_ [°C]
	Fe_3_O_4_	Fe	Fe [wt%]	—			—		
				Top	Bottom	Top/Bottom (ave. wt%)	Film	Corr.^a)^	
1.5M_0.75G_t	40.0	28.9	31.0	26	39	0.67 (33)	30	69	9
1.5M_0.38G_t	44.4	32.1	31.1	20	46	0.42 (32)	28	65	18
2.25M_0.75G_t	50.0	36.2	39.0	29	50	0.57 (40)	42	77	31
2.25M_0.38G_t	54.6	39.5	43.6	29	50	0.58 (40)	39	65	30
1.5M_0.75G_T	40.0	28.9	34.0	21	49	0.42 (35)	38	80	43
2.25M_0.75G_T	50.0	36.2	39.5	25	47	0.53 (36)	41	76	82

^a)^ Corr. **–** Corrected for Fe_3_O_4_ content.

The magnetic properties of the films are studied by SQUID magnetometry, Table 2. The saturation magnetization (*M*_s_) increases with Fe concentration from an average of 32 ± 5 emu g^−1^ for films prepared with 1.5 w/v%, to 41 ± 1 emu g^−1^ for those prepared with 2.25 w/v% MNPs. When those values are corrected for the measured Fe_3_O_4_ content (from ICP‐OES), the average values are 72 ± 8 and 73 ± 7 emu g^−1^, at low and high MNP content. The close similarity and the fact that the values are in the upper part of the expected range, for Fe_3_O_4_ nanoparticles prepared by co‐precipitation, shows that particle crystallinity is retained in the nanocomposite. This aspect and high particle content, while maintaining sufficient dispersion to avoid strong dipolar interactions that would suppress the hyperthermic response, are apparent in the exceptionally high temperature increases, Δ*T*, that are obtained, Table 2, see below.

**Figure 3:**
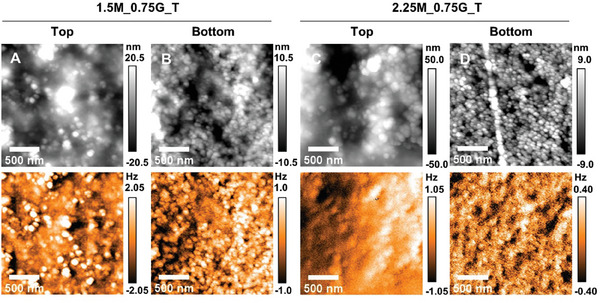
AFM (top row) and MFM (bottom row) images (2 × 2 µm) of A) top side of 1.5M_0.75G_T film, B) bottom side of 1.5M_0.75G_T film, C) top side of 2.25M_0.75G_T film, and D) bottom side of 2.25M_0.75G_T film. The MFM images were acquired with a 30 nm lift height. These images are representative of several scanned areas of the films.

The effect of MNP concentration on the topography and magnetic response of top and bottom sides of 1.5M_0.75_T and 2.25M_0.75_T films is investigated by atomic force microscopy (AFM) and magnetic force microscopy (MFM), **Figure** **3**. The topographic images clearly show MNPs at the surface of the films (seen as the brightest regions in MFM). Higher MNP concentration on the bottom side of the films, apparent from EDS, is also observed. The root mean square (RMS) roughness values of the 1.5M_0.75_T film are 10.2 ± 0.3 nm (top) and 6.3 ± 0.8 nm (bottom), and 17.1 ± 1.1 nm (top) and 7.1 ± 2.7 nm (bottom) in the 2.25M_0.75_T film; see Figure 3A–D, respectively. Increased roughness of the top surface of 2.25M_0.75_T, as compared to 1.5M_0.75_T, is attributed to higher MNP concentration present in the former film. The lower roughness of the bottom sides is also due to the confinement of the bionanocomposite suspension in contact with the acrylic mold during solvent casting, consistent with the presence of liquid glycerol late in the drying as suggested by SEM. The line observed in the lower topography image of the 1.5M_0.75_T film is an imprinted scratch from the casting mold, Figure 3D. The AFM and MFM images shown in Figure 3 correspond exactly to the same location. The repulsive (attractive) magnetic force gradient between tip and sample shifts the resonance curve of the tip to higher (lower) frequencies, which corresponds to the bright (dark) contrast areas. The topography of the films is dominated by the MNPs, which is also responsible for the magnetic response. Therefore, a correlation between the topography and MFM frequency images is observed in some areas. The MFM images show a homogenous dispersion of MNPs throughout the chitosan matrix. In contrast, the topography of the control chitosan–glycerol only film is flat; RMS value of 2.1 ± 0.1 nm and MFM imaging show no signal; Figure S2, Supporting Information. Overall, the film characterization (SEM, EDS, ICP‐OES, magnetometry, AFM and MFM) demonstrates the formation of films that are consistent in the lateral dimension with a linear gradient of MNP incorporation.

### 2.3 Spatiotemporally Resolved Hyperthermia by Situ LC‐AMF/Video Thermography

The hyperthermic efficiency, or specific loss power (SLP), of the aqueous MNP suspension is previously determined (at *ν*_AC_ 276 kHz, H_AC_ 14.7 kA m^−1^) to be 30 W g^−1^,^[^ ^26^ ^]^ corresponding to an intrinsic loss power (ILP) of 0.5 nH m^2^ kg^−1^. The measured SLP value for film 2.25M_0.75_T immersed in cellular medium under the same measurement conditions is 34 W g^−1^, corresponding to an ILP of 0.6 nH m^2^ kg^−1^. The heating response of the film in cellular medium is shown in Figure S3, Supporting Information. As the MNPs are immobilized in the chitosan matrix, it is expected that the Brownian relaxation contribution will be completely suppressed for the films; and hence, the ILP reduces as only the Néel contribution remains. The similar ILP value observed for the aqueous MNP suspension strongly suggests reduced Brownian motion of the particles in this case, presumably due to aggregation. Note that the DLS response of the suspensions under these conditions is not stable, with fluctuating high size, with *Z*_ave_ 200–760 nm and polydispersity index, PDI, ≥0.23; both values progressively increase over periods of days.

The hyperthermia response of bionanocomposite films was evaluated in situ using a thermal camera and an open AMF irradiation coil (LC‐AMF) that accommodated a standard 35 mm diameter Petri dish.^[^ ^5^ ^]^ All the measurements were undertaken with the top part of films (lower MNP content) upward. The camera provided the temperature changes of the top surface of any sample. The starting temperatures were in the range 18–22 °C (lab temperature); any initial dissimilarity was minor compared to the typical temperature increases, Δ*T*, achieved. Note that for this reason, while absolute temperatures were shown in the figures; Δ*T* values were discussed in the text. The conditions used for thermography (*ν*_AC_ 664.2 kHz, H_AC_ 12.8 kA m^−1^) were within the established clinical range.^[^ ^40^ ^]^

**Figure 4:**
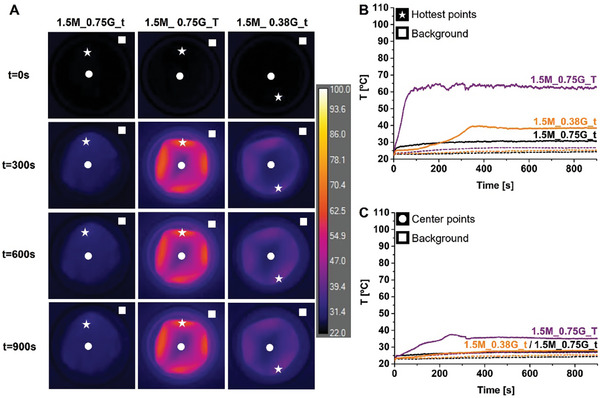
A) LC‐AMF thermal images (*xy*‐plane) from the top surface of films containing 1.5 w/v% magnetite at *t* = 0, 300, 600, and 900 s, placed on a glass Petri dish in the LC‐AMF setup. Measurements were performed at magnetic field strength *H*_AC_ 12.8 kA m^−1^ and frequency *ν*_AC_ of 664 kHz. B) Evolution of temperature as a function of time at the hottest points (white stars, full line), and the corresponding background points (white squares, dashed lines). C) Evolution of temperature as a function of time at the center points (white circles, full lines) and the corresponding background points (white squares, dashed lines).

**Figure 5:**
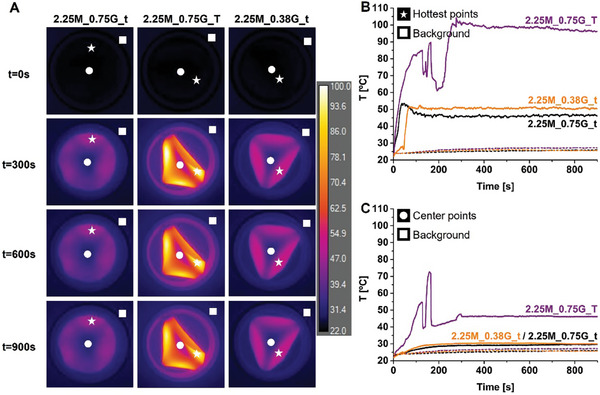
A) LC‐AMF thermal images from the top surface of films containing 2.25 w/v% magnetite at *t* = 0, 300, 600, and 900 s, placed on a glass Petri dish in the LC‐AMF setup. Measurements performed at *H*_AC_ 12.8 kA m^−1^ and *ν*_AC_ 664.2 kHz. B) Evolution of temperature as a function of time at the hottest points (white stars, full line) and the corresponding background points (white squares, dashed lines). C) Evolution of temperature as a function of time at the center points (white circles, full lines) and the corresponding background points (white squares, dashed lines).

The heating of films in Petri dishes, with 1.5 w/v% and 2.25 w/v% nominal MNP content and without any liquid added, over 900 s irradiation time is shown in **Figures** **4** and **5**, respectively. We observed significant spatial differences in the induced heating of all films, which mostly progressed “outside‐in,” that is, from the border toward the middle of the films, Figures 4 and 5A; Figures S4 and S5A, Supporting Information. The films reached a temperature plateau within 100–120 s, Figure 4B,C and 5B,C, when surface heat losses balanced the magnetic energy input. In some cases, temperature fluctuations were observed which were associated with heat‐induced folding, Figures 4A and 5A, discussed below. The induced heating response was checked in several cases and was found to be reproducible over at least three cycles without changes to film functionality and with no apparent particle leaching. Aside from folding, there was no apparent water loss or other change in the films on irradiation. Chitosan‐only films did not show any heating on AMF irradiation, Figure S6, Supporting Information.

The Δ*T*_max_ values for all the films studied are included in Table 2, which is arranged in order of increasing MNP mass in the films, as determined by ICP‐OES. It is immediately clear that MNP mass is the strongest predictor of Δ*T*_max_. The thin films, prepared at lower MNP content, 1.5M_0.75G_t and 1.5M_0.38G_t, with the same outer surface area (and hence intrinsic heat dissipation) and total Fe (from ICP‐OES), achieve a temperature at the plateau, Δ*T*_max_, of 9 °C and 18 °C, respectively, Figure 4A,B, Table 2. The higher glycerol sample is more homogeneous from the viewpoint of vertical MNP distribution (0.67 top/bottom Fe (wt%) ratio, as compared to 0.42). The difference between the Δ*T*_max_ achieved may be assigned to higher MNP content in the second sample. Similarly, the thicker sample, 1.5M_0.75G_T, induces a stronger response, with the higher Δ*T*_max_ of 43 °C (at 50 s) than is observed for 1.5M_0.75G_t (thinner film of the same formulation). We ascribe this to higher total MNP mass in the film.

For thin films prepared at higher MNP content, 2.25M_0.75G_t and 2.25M_0.38G_t, similar Δ*T*_max_ values of 31 °C and 30 °C are observed, that are significantly higher than for the two lower MNP content samples; again these films have very similar top layer MNP content. The strongest heating is verified for the thick film, 2.25M_0.75G_T, with a Δ*T*_max_ of 82 °C after ≈100 s, as expected, given this is the film of highest MNP mass, Figure 5; Movie S1, Supporting Information.

These results demonstrate that the key design parameter enabling tuning of hyperthermic response is the MNP mass, which can be controlled through MNP concentration and film thickness. While further optimisation of composition/response is possible, the temperature increases achieved after ≈100 s exceed the required temperatures for magnetic hyperthermia (41–45 °C) enabling use of shorter irradiation times or lower field strengths.^[^ ^10^ ^]^ A key aspect is that precise temperature control can be achieved at the surface with AMF stimulus. Further, the exceptional heating of the partially optimised 2.25M_0.75G_T film, reaching 104 °C, Figure 5B, can be useful for sterilisation purposes using the AMF prior to biomedical applications.

The glass Petri dish used to hold the films in the field, and to retain medium see below, may contribute to heat dissipation and affect the outcomes. Experiments conducted placing the films directly on PTFE tape (a thermal insulator; Figures S4 and S5 and Video S2, Supporting Information) within the LC‐AMF, as compared to in the Petri dishes (Figures 4 and 5; Video S1, Supporting Information) resulted in higher induced temperature increase within the films, as is clear from the two movies. In this case 1.5M_0.75G_T, 1.5M_0.38G_t and 1.5M_0.75G_t achieved Δ*T*_max_ of 57 °C, 25 °C, and 14 °C, respectively, Figure S4, Supporting Information. The higher MNP content films, 2.25M_0.75G_T, 2.25M_0.38G_t and 2.25M_0.75G_t, achieved Δ*T*_max_ of 75 °C, 41 °C, and 40 °C, respectively, Figure S5, Supporting Information. Petri dishes were used for the remainder of the study; while, the Δ*T*_max_ was typically lower, the heating was more consistent and was more than sufficient for the applications evaluated below. The films display superior heating efficiency than the magnetic bionanocomposites previously reported in the literature. For example, multi‐layered films composed of alginate/chitosan and MNP/chitosan layers achieved a Δ*T*_max_ of 12 °C after 300 s under AMF (*ν*_AC_ 180 kHz, *H*_AC_ 35 kA m^−1^).^[^ ^34^ ^]^ Similarly, MNP/alginate/polyvinyl alcohol mats achieved a Δ*T*_max_ of 15 °C after 110 s under AMF (≈750–1150 kHz and 6.4 kW).^[^ ^35^ ^]^

The greater induced heating and folding observed at the edges of the films, on exposure to the field in the dry state, are interesting and informative. Our previous studies on magnetic hydrogels reveal that for homogeneous MNP‐loaded disks, the heating induced by this LC‐AMF instrument is uniform across the sample; hence, the AMF is considered homogeneous.^[^ ^5^ ^]^ In addition, the films have homogenous lateral (*xy*) MNP dispersion, as demonstrated by EDS and AFM/MFM; Table 2 and Figure 3, respectively. All the films show some folding, apart from 1.5M_0.75G_t, which only reaches Δ*T*_max_ of 14 °C; Figure S4A, Supporting Information. In contrast, 2.25M_0.75G_T with Δ*T*_max_ of 75 °C rolled into a tube; Figure S5A and Video S2, Supporting Information. The extent of folding is dependent on the temperature increase. The folding is reversible; when the AMF is removed the films return to their original flat position and any samples that are checked retain their heating efficiency. Folding lifts the films, breaking local contact with the glass of the Petri dish, which reduces heat dissipation. Consequently, the highest temperatures achieved by the films are observed at the edges.

Folding has been reported for chitosan‐based films when either in contact with water or exposed to solvents due to rapid local dehydration.^[^ ^33^, ^41^ ^]^ We suggest that the induced temperature increase in the films due to the MNPs promotes internal water flux in the *z*‐(vertical) direction, reducing the water content at the lower part of the film (higher MNP content). However, there is no evidence of water accumulation on, or loss from, the upper surface, and as noted, the effect is fully cyclable. The water concentration gradient leads to a strain gradient; and hence, folding for unrestrained films. This interpretation is further supported by the observation that placing the 2.25M_0.75G_T film in ethanol induces folding, and on placing it in water, that the film flattens back out; Video S3, Supporting Information. This effect is not seen for the equivalent non‐magnetic film, suggesting that the mechanical effects are exacerbated by the *z*‐gradient in MNP content; Video S3, Supporting Information. AMF heating‐induced folding and rolling may be interesting for applications in soft magnetic robotics.^[^ ^42^, ^43^ ^]^ The patterning of films with MNPs; for example, using screen‐printing processes,^[^ ^44^ ^]^ could be used to prepare materials with controlled shape changes under AMF.

### 2.4 Use of Spatiotemporal Hyperthermia in Biomedical Settings

The bionanocomposite films were designed for MH cancer therapy. Hence; in this section, we describe a preliminary evaluation of the performance of the 2.25M_0.75G_T film because it reveals the strongest AMF‐induced heating. This includes the evaluation of the spatiotemporally resolved hyperthermic response of the film when in contact with cell culture medium, its use for contact heating of hydrogels, and its utility for MH treatment of MNT‐1 human melanoma cells.

#### 2.4.1 In Situ LC‐AMF With Cell Culture Medium

**Figure 6:**
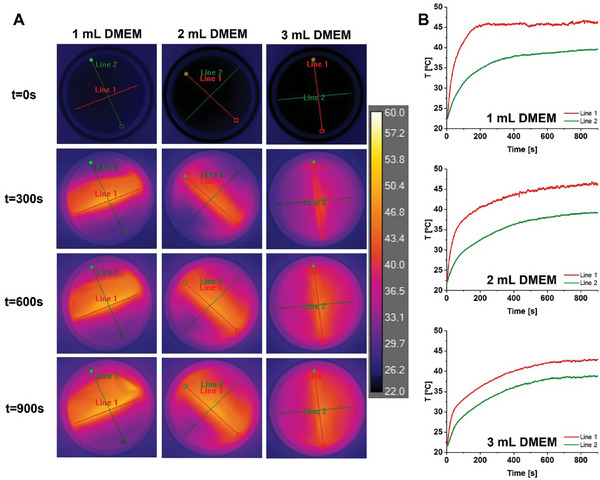
A) LC‐AMF thermal images from the top surface of 2.25M_0.75G_T film at *t* = 0, 300, 600, and 900 s, placed on a glass Petri dish and covered with 1, 2, and 3 mL of DMEM cell culture medium. Measurements were performed at a magnetic field strength of *H*_AC_ 12.8 kA m^−1^ and *ν*_AC_ 664 kHz. B) Evolution of temperature as a function of time at the hottest points along the lines indicated.

The 2.25M_0.75G_T was subsequentially covered with 1, 2, or 3 mL of DMEM, Figure S7, Supporting Information, and exposed to the AMF after each DMEM addition. LC‐AMF data for 2.25M_0.75G_T in contact with initially 1 mL DMEM cell culture medium is shown in **Figure** **6**. Folding of the film was observed immediately on addition of DMEM, consistent with the previous observations. Despite folding, the film efficiently heated on exposure to AMF, with the liquid surface rapidly achieving an average Δ*T*_max_ of 24 °C within 200 s (along the line of highest temperature, Line 1, directly above the center of the film). There was a 7.5 °C drop in temperature from the center to the tips of Line 1, even after 900 s; Figure S8, Supporting Information, with a concave dependence of temperature on distance from the center (i.e.*, d*T/*d*x increasing further out). Along Line 2 (perpendicular to Line 1), the Δ*T*_max_ of 17 °C was more gradually achieved. There was a 13.5 °C drop in temperature from the center to the tips of Line 2 at 900 s. In this case, the dependence on distance from the center was convex. Clearly there was rapid transport of heat upward; and apparently, slower subsequent heating of the volume of medium.

Also included in Figure 6 are experiments in which a further 1 mL (total 2 mL in glass dish) medium was then added to the same sample before AMF exposure; and then, a further 1 mL (total 3 mL in glass dish) before exposure once again. Photographic images, Figure S7, Supporting Information, show that the extent of film folding was unchanged on adding more DMEM. The thickness of the strongly responding part of the upper reflective surface became progressively narrower than the width of the submerged film as the volume of DMEM and distance to the upper liquid surface increased. The maximal heating above the film decreased very weakly, but it took significantly longer to reach the plateau with added medium, with ∆*T*_max_ along Line 1 of 24 °C (in 200 s), 24 °C (in 800 s), and 22 °C (in 800 s), for 1, 2, and 3 mL DMEM, respectively. The dependence of temperature on distance from the center of Line 1 was again concave. Along Line 2, the difference in temperature was far higher, as expected, and after 600 s, settled at 14 °C, 13 °C, and 8 °C, for 1, 2, and 3 mL DMEM, respectively. The convex dependence of temperature on distance along the line from the center was maintained, and the profiles were very similar in shape. Interestingly, for all volumes of DMEM added, the initial temperature jumps (*dT/dt*)*_t_*_= 0→30s_ on Line 1 were identical, within error. This clarifies that initially there was a “wedge” of the DMEM directly above the film, which became thinner further from the film surface, in which there was very rapid transport of heat upward and from which there was weaker lateral transfer. With increasing DMEM, due to increased total dissipation to the sides, this “wedge” became progressively narrower. On the other hand, the heating profile off‐feature (Line 2) was far less sensitive to the medium volume and the Δ*T* values were almost identical for 2 and 3 mL over the 900 s studied.

#### 2.4.2 Inducing Heating in Soft Hydrogels

**Figure 7:**
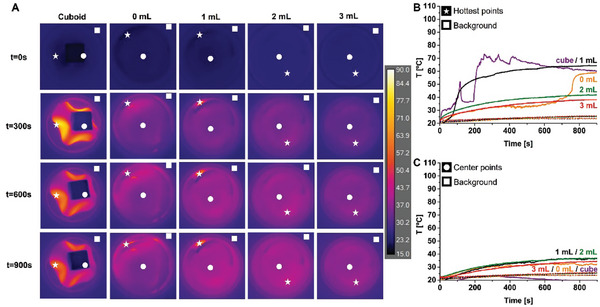
LC‐AMF thermography of the 2.25M_0.75G_T film supporting 10 w/v% mTG crosslinked gelatine in different formats. A) Thermographic images recorded (from the top) at *t* = 0, 300, 600, and 900 s for from left; “cuboid” a 0.6 mL gelatin cuboid with no medium and no parafilm layer; ‘0 mL’ a thin layer of gelatin (0.6 mL filling the 35 mm wide petri dish to a height of 0.63 mm) on top of a single layer of parafilm that was placed over the film; “1–3 mL” the same thin layer of gelatin with added DMEM medium (filling the petri dish to an additional height of 1.04, 2.08, and 3.12 mm). Measurements were performed at *H*_AC_ 12.8 kA m^−1^ and *ν*_AC_ 663 kHz. The evolution of temperature as a function of time at B) the hottest points (full line, and white stars in A), and at C) the center points (full lines, white circles in [A]), the corresponding background points (dashed lines, white squares in [A]) are included.

We fabricated gelatine‐based hydrogels using enzymatic crosslinking of bacterial transglutaminase (mTG), named here mTG‐Gels. Gelatine was selected for the matrix as it is commonly applied in tissue engineering,^[^ ^45^ ^]^ and mTG as it is biocompatible / minimally cytotoxic. First, we examined the response of a cuboid‐shaped sample (10 w/v% gelatine 2% mTG, of 0.6 mL volume; *h* × *w* × *d*, 6 × 10 × 10 mm) placed directly on the magnetic film without medium, cuboid in **Figure** **7**A. Due to AMF‐induced mechanical changes within the film during irradiation, it acted as a soft robot pushing the cuboid upward; see Video S4, Supporting Information. Nevertheless the Δ*T*_max_ on the upper surface of the cuboid was 6 °C, over 900 s, Figure 7C. Note the thermographic change was not visually obvious in the image, but the color did change consistently across the square from black to indigo. Considering the gel was 6 mm thick, the induced upper surface heating was encouragingly strong and in line with the temperature jump needed to generate measurable changes in diffusion of low molecular weight species, for example, for dyes, as shown in our previous study.^[^ ^5^ ^]^ The temperature change on the film itself, Δ*T*_max_ of 54 °C, that is, to the sides of the cuboid, was strong, as expected, and it fluctuated significantly due to motion of this part of the film; although, this did not obstruct the thermography.

Folding would not be an issue for some applications, for example, as functional hyperthermic melanoma plasters in which case films would be retained flat. However, it could be limiting in some scenarios. By placing a single layer of parafilm directly on the top of the film, we were able to prevent folding on subsequent addition of medium. Therefore, for evaluating hydrogel response in medium, a single sheet of parafilm was placed over the film; and subsequently, a layer of mTG‐Gel (0.60 mL of 10 w/v% that filled the dish to 0.63 mm; see Experimental Section) was added. As expected, folding was suppressed in comparison with the experiments in the absence of parafilm, Figure S9, Supporting Information. Significant heating was observed across the hydrogel surface; see 0 mL in Figure 7B,C; Video S5, Supporting Information, with ∆*T*_max_ of 41 °C and ∆*T*_center_ of 14 °C. Addition of DMEM medium on top of the hydrogels resulted in temperature jumps ∆*T*_max_ of 46 °C, 19 °C, and 16 °C, for 1, 2, and 3 mL of DMEM added. However, the ∆*T*_max_ value of 46 °C seemed to correspond to an area that was not fully covered at that volume of medium. The ∆*T*_center_ values, where the films were evenly covered, were 18 °C, 15 °C, and 13 °C, respectively, Figure 7C. The temperature jumps clearly reduced with increasing DMEM volume and were lower than those shown in Figure 6, due to additional parafilm and gel content between the film and upper reflective surface. Between 1 and 2 mL, that is, 1.04 and 2.08 mm gel thickness, the inhomogeneities were flattened out and sufficient ∆*T* for biomedical applications was retained. The results demonstrate the possibility of effective use of magnetic films as heat mediators of soft constructs, which could be potentially used as cargo depots.^[^ ^46^ ^]^

#### 2.4.3 Hyperthermic Eradication of MNT‐1 Melanoma Cells

**Figure 8:**
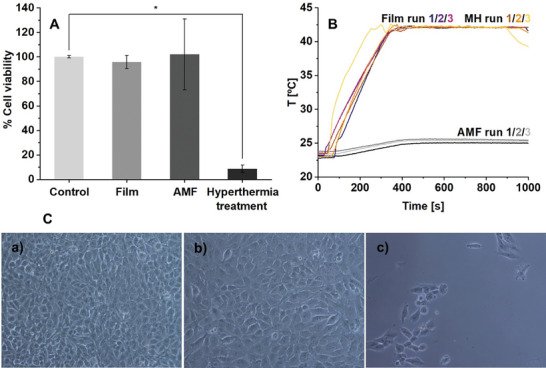
A) MNT‐1 cell viability percentage 48 h after: exposure to 2.25M_0.75G_T film extract (Film), AMF conditions (AMF), and hyperthermia treatment in the presence of 2.25M_0.75G_T film (Hyperthermia treatment). B) Heating curves of control without film (AMF), control sample without cells (Film), and with MH treatment to MNT‐1 cells. C) Light microscopy images of C‐a) MNT‐1 cells control sample, C‐b) MNT‐1 cells after AMF exposition, and C‐c) MNT‐1 cells after hyperthermia treatment with 2.25M_0.75G_T film. Measurements were performed at *H*_AC_ 11.2 kA m^−1^ and *ν*_AC_ 275 kHz. Statistical analysis: *: *p* < 0.05 is statistically different. Three independent replicates per sample were evaluated.

The cytotoxicity of the 2.25M_0.75G_T film to MNT‐1 melanoma cells was first evaluated by incubating cells with film extract over 48 h. The subsequent MTT assay showed no effect on cell viability, **Figure** **8**A (Film). In our previous work, a chitosan film containing 50% rGO‐Fe_3−_ *_x_* O_4_ also showed low toxicity toward the HaCat cell line. Similarly, AMF treatment (without film) had no measurable effect on viability; while, the application of AMF in the presence of 2.25M_075G_T resulted in cell death. These results suggest minimal baseline cytotoxicity of the magnetic bionanocomposite films, suggesting suitability for biomedical applications.^[^ ^36^ ^]^

Hyperthermia tests were performed using the setup shown schematically in Figure S10, Supporting Information. 2.25M_0.75G_T films were used in flasks containing DMEM cell culture medium and MNT‐1 cells supported on a coverslip on the bottom. The flask was exposed to an AMF; in this case of *ν*_AC_ 275 kHz and H_AC_ 14 kA m^−1^, which again was within the established clinical range.^[^ ^40^ ^]^The AMF heating curves for experiments without film and with MNT‐1 cells (AMF run), with film and without cells (Film run), and with film and cells (MH run) were displayed, for *n* = 3 repeats each, in Figure 8B. For both the MH and Film runs (both using 2.25M_0.75G_T), the film reached the stable target hyperthermia temperature of 42 °C within only 300 s. This short time to achieve the hyperthermic temperature was only possible due to the high heating efficiency of the film, that was retained when submerged in liquid medium, as shown in the previous sections. The AMF and temperature were maintained for 10 min; see Experimental Section. Identical heating curves of replicates confirmed again the homogeneity of films and the consistency and reproducibility of film response in the experimental setup. These characteristics are necessary for comparison of responses of different cell types, and rapid heating is critical for the same purpose as it provides defined times at the hyperthermic temperature.

The AMF run (no film) showed a marginal temperature increase and, as noted above, this condition had no significant effect on cell viability. Light microscopy photographs of cells taken after 48 h incubation (before starting the cell viability assay), Figure 8Ca, showed minimal influence of the AMF on cell morphology and confluence compared to the control, Figure 8Cb. On the other hand, the hyperthermia tests in the presence of the films drastically decreased cell viability to below 10%, and reduced cell number and morphological changes were apparent, Figure 8Cc. These effective reductions in MNT‐1 cell viability were achieved using only one short, 10 min, MH cycle with the partially optimized 2.25M_0.75G_T film. The data clearly demonstrates the effectiveness of the MH treatment and suggests high translational potential for the magnetic bionanocomposites.

## 3 Conclusion

A facile approach is presented to synthesize biocompatible chitosan‐based magnetic bionanocomposite films, with thickness < 100 µm, showing extraordinarily high and reproducible AMF‐induced temperature increases, reaching up to a maximum temperature increase of 82 °C at the surface of dry films. Video thermography during AMF exposure revealed key aspects of film performance for MH applications when in contact with hydrogels or placed within liquid medium over a significant submerged volume. The heat capacity of the surroundings did not limit heat flux directly above the film. With increasing volume of either medium or hydrogel, lateral heat transfer progressively increased and limited the temperature achieved. Homogeneous responses in the range needed for MH were easily accessible for the MNP concentrations described. Significantly stronger responses at the film; and hence, across larger medium volumes, should be possible through further formulation and particle optimization, opening out scope for applications. This study also provides valuable insights for downstream finite element modeling analysis and prediction of responses. These would be of particular value for understanding complex heat transfer processes in developing patterned chitosan‐based magnetic films.

The superior heating ability of 2.25M_0.75G_T was used to demonstrate the potential of the films for different scenarios relevant across biomedicine. The films could be used as substrates to induce appropriate heating responses in cell culture medium and in gels of different shapes. The eradication of MNT‐1 cells was accomplished with a single MH cycle, with scope to increase the number of treatment cycles. The high temperatures reached by the film could also be used for self‐sterilization. Film folding induced in response to the generation of an internal water concentration gradient may provide mechanical responses applicable in the development of self‐propelling devices, soft robots, and actuators.

## 4 Experimental Section

### Chemicals

Iron (III) chloride hexahydrate ≥ 98.0% (FeCl_3_.6H_2_O) was purchased from Merck KGaA. Ammonia hydroxide solution puriss. p.a. (NH_4_OH) was purchased from Honeywell Fluka. Iron (II) sulfate heptahydrate ≥ 99.0% (FeSO_4_·7H_2_O), chitosan (C_56_H_103_N_9_O_39_, medium molecular weight and ≥75% degree of deacetylation), and dimethyl sulfoxide were purchased from Sigma–Aldrich. Acetic acid glacial ≥  99.8% (CH_3_COOH) was purchased from Chem‐Lab NV. Glycerol 99.5% (C_3_H_8_O_3_) was purchased from Scharlau. Gelatine from porcine skin, gel strength 300, Type A was purchased from Merck. All chemicals were analytical grade and used as received.

### Synthesis of MNPs

MNPs were synthesized by a co‐precipitation method.^[^ ^37^ ^]^ Distilled water (75 mL) was deoxygenated by bubbling nitrogen for 30 min, followed by the dissolution of the iron precursors, FeCl_3_·6H_2_O (3.77 g) and FeSO_4_·7H_2_O (3.20 g), that remained under stirring for additional 30 min. Finally, the NH_4_OH (10 mL) was added, and a black precipitate was immediately formed. The nitrogen flow was maintained during all the reaction. The product was magnetically separated and washed with distilled water until a neutral pH was reached.

### Preparation of Magnetic Films

The bionanocomposite films were prepared using a procedure adapted with minor changes from the authors’ previous work.^[^ ^36^ ^]^ Magnetite (1.5 w/v% or 2.25 w/v%) in dry form was dispersed by stirring in a 0.1 m acetic acid solution. The speciation of Fe hydroxide with pH is well known (a weak excess positive surface charge is expected under these conditions).^[^ ^47^ ^]^ Chitosan (1.5 w/v%) was then added to the dispersion and allowed to dissolve overnight at room temperature (≈20 °C). The bionanocomposite solution was vacuum filtrated using a nylon cloth. Glycerol plasticizer (0.75 w/v% or 0.38 w/v%) was added and homogenized by stirring at 50 °C for 10 min. Different amounts of the solution (0.2 or 0.4 g cm^−2^) were transferred to poly(methyl methacrylate) molds (hereafter designed by “acrylic molds”) (6 × 6 cm^2^) to obtain films of different thicknesses (defined in main text as thin or thick, respectively). The solutions were dried in a ventilated oven overnight at 35 °C to obtain the films by solvent casting. The composition and thickness of the bionanocomposite films are shown in Table 1. The different samples were named as following: *x*M_*y*G_t/T where *x* and *y* stand for magnetite (M) and glycerol (G) concentrations (w/v%), respectively, followed by the information regarding the films thickness, with *t* and *T* denoting thin and thick films, respectively.

### Preparation of Microbial Transglutaminase (mTG)‐Crosslinked Gelatine Hydrogels

Type A, 300 bloom porcine gelatine (G2500, Sigma–Aldrich, Merck) at 8 w/v% or 20 w/v% and microbial transglutaminase (mTG, 1000–100Activa RM Transglutaminase – 100 g, Ajinomoto, Tokyo, Japan) at 4 w/v% were dissolved separately in HPLC‐grade water at 37 °C until clear liquids with no bubbles were obtained. Both solutions (pre‐warmed at 37 °C) were then gently mixed in equal volumes (50:50%) to make hydrogels with final concentrations of 4 w/v% or 10 w/v% gelatine and 2 w/v% mTG.

The gels took the shape of the container they were crosslinked in, so as to obtain:

- A gel cuboid of 0.6 cm^3^: 0.6 mL of the mixed mTG and gelatine solutions were pipetted into polystyrene macro cuvettes for visible wavelengths (Fisher) to form gels with dimensions 6  ×  10  ×  10 mm (h  ×  w  ×  d). After successfull crosslinking, the gels were gently removed from the cuvettes and used immediately for LC‐AMF experiments.
- For thin gel films: 0.5 or 1.0 mL mixed mTG and gelatine solutions were pipetted into glass Petri dishes of dimensions 29  ×  12 mm (d  ×  h) to obtain circular films of thickness 0.76 and 1.51 mm, respectively. All films were pipetted on top of the parafilm tape of the same circular diameter as the petri‐dish.

### Characterization of MNPs and Bionanocomposite Films

*X‐Ray Diffraction (XRD)*: The crystalline structure of MNPs was investigated in reflection mode using a Panalytical Empyrean X‐ray diffractometer with Cu Kα radiation (*λ* = 1.5418 Å), a step size of 0.0260° (2*θ*), and a scan step time of 96.3900 s. The XRD patterns were analyzed using the powder diffraction file (PDF) database of the International Center for Diffraction Data (ICDD).

*Transmission Electron Microscopy (TEM)*: The morphology, size, and dispersion of MNPs were characterized by TEM using a HRTEM‐200 equipment (JEOL, model 2200FS). MNPs were dispersed in distilled water until transparent suspensions were obtained. The particle size distribution was obtained measuring the diameter of 300 MNPs from different TEM images using the ImageJ software.^[^ ^48^ ^]^ The histogram was fitted assuming a Log‐Normal distribution curve using OriginPro 9.0 software.

*Zeta Potential*: The surface charge and stability of magnetite in aqueous solution were investigated by zeta potential. The pH of the solution was measured using a CONSORT P800 pH meter, being adjusted with 0.001, 0.01, 0.1, and 1 m HCl and NaOH. The measurements were performed on Zetasizer Nano ZS equipment (Malvern).

*Thickness of Films*: The thickness was measured using a digital micrometer (Mitutoyo Corporation). Five measurements (*n* = 5) of different locations of each film were performed.

*Scanning Electron Microscopy (SEM) and Energy‐Dispersive Spectroscopy (EDS)*: The microstructure and elemental composition were studied using a SEM SU‐70 microscope (Hitachi SU‐70, Tokyo, Japan) coupled with a Quantax 400 EDS (Bruker) detector. Bionanocomposite films were cut and stuck onto a double‐sided carbon tape in order to acquire top‐view images of both sides of each film (the side in contact with the acrylic mold during solvent casting processing was designated as “bottom” while the other was named “top”). Conductive carbon thin film was deposited onto the films using a carbon rod coater (Emitech K950X). The micrographs were acquired at an accelerating voltage of 15.0 kV and working distance of 15 mm using field‐free mode detection.

*Atomic Force Microscopy (AFM) and Magnetic Force Microscopy (MFM)*: The measurements were performed using a Cypher AFM (Asylum Research) and AFM probes with CoCr tip coating and nominal spring constant of 2.8 N m^−1^ and resonance frequency of 75 kHz (ASYMFMHM, Asylum Research). The probes were magnetized prior to imaging by placing the probe on a permanent magnet before loading it into the cantilever holder. The MFM is a two‐pass technique; first, the topography is acquired and second, the magnetic interactions between the magnetized tip and sample are evaluated by lifting the tip off the surface.^[^ ^49^ ^]^

*Inductively Coupled Plasma – Optical Emission Spectrometry (ICP‐OES)*: The films (10 mg) were digested in 5 mL of 1% nitric acid at 60 °C for 1 h using a sonication bath. The iron was quantified using an Optima 4300 DV spectrometer (Perkin Elmer) with a resolution of 0.006 nm at 200 nm. The analyses were performed at C.A.C.T.I. center, Vigo – Spain.

*Magnetic Properties*: The magnetization measurements were carried out using a superconducting quantum interference device (SQUID) magnetometer Quantum Design MPMS2. Magnetic moment measurements were carried out as function of increasing temperature after cooling in zero magnetic field (zfc) or in the measurement field (fc) of 5 mT and, for chosen temperatures, as a function of magnetic field up to 5.5 T.

*In Situ Thermal Imaging During Alternating Magnetic Field (LC‐AMF) Irradiation*: Measureme3nts on bionanocomposite films were carried out on a live cell–alternating magnetic field (LC‐AMF) module connected to a NanoTherics NAN201003 MagneTherm system which enables RF irradiation of a sample placed in a standard Petri dish (of diameter *d* = 35 mm). Measurements were carried out at a frequency of 663 kHz or 664.2 kHz and at field strength of 12.8 kA m^−1^. Thermal imaging was performed using a Flir A655sc thermal camera (Flir, Butler Technologies). The camera was aligned to view films carefully placed either in: i) 35 mm glass Petri dishes or ii) placed directly on polytetrafluoroethylene (PTFE) tape in the LC‐AMF setup. Recordings were made for 15 min and the AMF was turned on immediately after the recording started. Each 15 min recording consisted of 5625 frames at 6.25 Hz. The resolution of the A655sc camera was ≈170 µm (pixel size). The whole setup was placed in a dark room to minimize IR reflections and temperature fluctuations during imaging. Temperature calibration up to 150 °C was auto performed. The internal calibrations or Non‐Uniformity Corrections, accorded to ISO9001:2008 were as detailed at https://www.flir.eu/browse/oem-cameras-components-and-lasers/thermal-camera-cores/.

For cell culture heating experiments, Dulbecco's modified Eagle medium (DMEM) supplemented with 10% fetal bovine serum (FBS), 100 units mL^−1^ penicillin, 1 mg mL^−1^ streptomycin, and 2 mm L‐glutamine were used and carefully pipetted on the top of the films in a noted volume (1, 2, or 3 mL). For hydrogels heating experiments, hydrogels (either as a gel cuboid or thin film) were placed directly on top of the magnetic bionanocomposite films previously placed in a glass Petri dish of 35 mm diameter.

*Cytotoxicity of 2.25M_0.75G_T Film to MNT‐1 Melanoma Cells*: The MNT‐1 melanoma cells were cultured in DMEM culture medium and incubated at 37 °C in 5% CO_2_ humidified atmosphere. The 2.25M_0.75G_T film cytotoxicity was evaluated by the exposure of film extract to MNT‐1 cells. Film pieces with 1 × 1 cm^2^ were sterilized in ethanol 70% during 1 h; and then, rinsed several times in sterile ultra‐pure water and finally rinsed in sterile phosphate buffered saline (PBS). The sterilized film was incubated in 1 mL DMEM during 24 h at 37 °C and 5% CO_2_ to obtain the extract.^[^ ^36^ ^]^ Three extract replicates were prepared. MNT‐1 cells were seeded in a 96 well plate and allowed to adhere. After 24 h, the DMEM culture medium was replaced by the film extracts and the cells were incubated during 48 h. The cells viability was evaluated by the 3‐(4,5‐dimethyl‐2‐thiazolyl)‐ 2,5‐diphenyl tetrazoliumbromide (MTT) assay.^[^ ^50^ ^]^ Briefly, 50 µL of 1 g L^−1^ MTT solution was added to each well. After 4 h of incubation, the medium was replaced by 150 µL of dimethyl sulfoxide and the plate stirred for 2 h. The absorbance was read at 570 nm using a microtiter plate reader (Synergy HT Multi‐Mode, BioTeK, Winooski, VT). Relative cell viability after exposure to the different extracts was calculated as percentage respective to control cells incubated with DMEM medium. Six replicates were used.

*Hyperthermia Tests*: The MNT‐1 cells were seeded in glass coverslips placed on 12 wells plates and cultured under the conditions described above. After 24 h of incubation, the coverslips were transferred to glass flasks adapted with a glass capillary tube on the lid to insert the temperature sensor (Photon‐control fiber optic sensor). The flasks were filled with 10 mL of DMEM, and a 3 × 3 cm^2^ piece of 2.25M_0.75G_T was placed inside each flask. Control samples without film and without cells were also evaluated. The hyperthermia tests were performed using an Easy Heat 0224 induction heating device (Ambrell) for 275 kHz frequency and 14 kA m^−1^ amplitude AMF. The flasks containing the liquid medium, film, and coverslip with cells were heated by magnetic induction from room temperature of ≈22 °C up to 42 °C, which was typically reached in ≈10 min. Afterward, the hyperthermic temperature of 42 °C was maintained during 10 min by controlling the amplitude of the AMF. After the hyperthermia tests, the coverslips were transferred to a 12 wells plate and incubated for 48 h. The cells viability was evaluated by the MTT assay, and the cells viability was calculated as described above.

*Statistical Analysis*: MTT cell viability assay data is expressed as mean ± standard deviation of at least three replicates. Data was analyzed by one‐way analysis of variance (ANOVA) and Tukey comparison test. Statistically significant values were considered if *p* value < 0.05. Thickness data is expressed as mean ± standard deviation of five replicates.

## Conflict of Interest

The authors declare no conflict of interest.

## Supporting information

Supporting Information

Supplemental Movie 1

Supplemental Movie 2

Supplemental Movie 3

Supplemental Movie 4

Supplemental Movie 5

## Data Availability

The data that support the findings of this study are available from the corresponding author upon reasonable request.

## References

[1] E. K. Fox , F. El Haddassi , J. Hierrezuelo , T. Ninjbadgar , J. K. Stolarczyk , J. Merlin , D. F. Brougham , Small 2018, 14, 1802278. 10.1002/smll.201802278 30589504

[2] J. Dulińska‐Litewka , A. Łazarczyk , P. Hałubiec , O. Szafrański , K. Karnas , A. Karewicz , Materials 2019, 12, 617. 30791358 10.3390/ma12040617 PMC6416629

[3] J. K. Wychowaniec , D. F. Brougham , Adv. Sci. 2022, 9, 2202278. 10.1002/advs.202202278 PMC9731717 36228106

[4] N. A. Jalili , M. K. Jaiswal , C. W. Peak , L. M. Cross , A. K. Gaharwar , Nanoscale 2017, 9, 15379. 28975171 10.1039/c7nr02327h PMC5702913

[5] P. Monks , J. K. Wychowaniec , E. Mckiernan , S. Clerkin , J. Crean , B. J. Rodriguez , E. G. Reynaud , A. Heise , D. F. Brougham , Small 2021, 17, 2004452. 10.1002/smll.202004452 33369876

[6] O. L. Lanier , O. I. Korotych , A. G. Monsalve , D. Wable , S. Savliwala , N. W. F. Grooms , C. Nacea , O. R. Tuitt , J. Dobson , Int. J. Hyperthermia 2019, 36, 687. 31340687 10.1080/02656736.2019.1628313

[7] P. A. Bottomley , E. R. Andrew , Phys. Med. Biol. 1978, 23, 630. 704667 10.1088/0031-9155/23/4/006

[8] P. J. Sugumaran , X.‐L. Liu , T. S. Herng , E. Peng , J. Ding , ACS Appl. Mater. Interfaces 2019, 11, 22703. 31244027 10.1021/acsami.9b04261

[9] J. Kolosnjaj‐Tabi , R. Di Corato , L. Lartigue , I. Marangon , P. Guardia , A. K. A. Silva , N. Luciani , O. Clément , P. Flaud , J. V. Singh , P. Decuzzi , T. Pellegrino , C. Wilhelm , F. Gazeau , ACS Nano 2014, 8, 4268. 24738788 10.1021/nn405356r

[10] A. Hervault , N. T. K. Thanh , Nanoscale 2014, 6, 11553. 25212238 10.1039/c4nr03482a

[11] N. K. Prasad , K. Rathinasamy , D. Panda , D. Bahadur , J. Mater. Chem. 2007, 17, 5042.

[12] M. B. Lodi , N. Curreli , S. Zappia , L. Pilia , M. F. Casula , S. Fiorito , I. Catapano , F. Desogus , T. Pellegrino , I. Kriegel , L. Crocco , G. Mazzarella , A. Fanti , IEEE Trans. Biomed. Eng. 2022, 69, 2029. 34882544 10.1109/TBME.2021.3134208

[13] F. Serio , N. Silvestri , S. Kumar Avugadda , G. E. P. Nucci , S. Nitti , V. Onesto , F. Catalano , E. D'amone , G. Gigli , L. L. Del Mercato , T. Pellegrino , J. Colloid Interface Sci. 2022, 607, 34. 34492351 10.1016/j.jcis.2021.08.153

[14] H. Gavilán , S. K. Avugadda , T. Fernández‐Cabada , N. Soni , M. Cassani , B. T. Mai , R. Chantrell , T. Pellegrino , Chem. Soc. Rev. 2021, 50, 11614. 34661212 10.1039/d1cs00427a

[15] H. Etemadi , P. G. Plieger , Adv. Ther. 2020, 3, 2000061.

[16] Vall d'Hebron enrolls the first patient in a clinical trialdesigned to treat locally advanced pancreatic cancer with nanoparticles. https://www.vallhebron.com/en/news/news/vall-dhebron-enrolls-first-patient-clinical-trial-designed-treat-locally-advanced-pancreatic-cancer-nanoparticles.

[17] RCL To Start Clinical Hyperthermia Study2021, www.resonantcircuits.com/news/clinical‐trial (accessed: July 2023).

[18] I. Castellanos‐Rubio , I. Rodrigo , A. Olazagoitia‐Garmendia , O. Arriortua , I. Gil De Muro , J. S. Garitaonandia , J. R. Bilbao , M. L. Fdez‐Gubieda , F. Plazaola , I. Orue , A. Castellanos‐Rubio , M. Insausti , ACS Appl. Mater. Interfaces 2020, 12, 27917. 32464047 10.1021/acsami.0c03222 PMC8489799

[19] G. Singh , N. Kumar , P. K. Avti , Int. J. Heat Mass Transfer 2020, 148, 119129.

[20] M. Sefidgar , E. Bashooki , P. Shojaee , J. Therm. Biol. 2020, 94, 102742. 33292983 10.1016/j.jtherbio.2020.102742

[21] C. Guibert , V. Dupuis , V. Peyre , J. Fresnais , J. Phys. Chem. C 2015, 119, 28148.

[22] S. E. Favela‐Camacho , E. J. Samaniego‐Benítez , A. Godínez‐García , L. M. Avilés‐Arellano , J. F. Pérez‐Robles , Colloids Surf., A 2019, 574, 29.

[23] V. F. Cardoso , A. Francesko , C. Ribeiro , M. Bañobre‐López , P. Martins , S. Lanceros‐Mendez , Adv. Healthcare Mater. 2017, 7, 1700845. 10.1002/adhm.201700845 29280314

[24] B. Pelaz , P. Del Pino , P. Maffre , R. Hartmann , M. Gallego , S. Rivera‐Fernández , J. M. De La Fuente , G. U. Nienhaus , W. J. Parak , ACS Nano 2015, 9, 6996. 26079146 10.1021/acsnano.5b01326

[25] E. Amstad , T. Gillich , I. Bilecka , M. Textor , E. Reimhult , Nano Lett. 2009, 9, 4042. 19835370 10.1021/nl902212q

[26] J. Gonçalves , C. Nunes , L. Ferreira , M. M. Cruz , H. Oliveira , V. Bastos , Á. Mayoral , Q. Zhang , P. Ferreira , Nanomaterials 2021, 11, 2939. 34835704 10.3390/nano11112939 PMC8623727

[27] Y. González‐Alfaro , P. Aranda , F. M. Fernandes , B. Wicklein , M. Darder , E. Ruiz‐Hitzky , Adv. Mater. 2011, 23, 5224. 21913233 10.1002/adma.201101364

[28] D.‐I. Kim , H. Lee , S.‐H. Kwon , Y. J. Sung , W. K. Song , S. Park , Adv. Healthcare Mater. 2020, 9, 2000118. 10.1002/adhm.202000118 32431072

[29] A. Augurio , P. Cortelletti , R. Tognato , A. Rios , R. Levato , J. Malda , M. Alini , D. Eglin , G. Giancane , A. Speghini , T. Serra , Adv. Intell. Syst. 2020, 2, 1900105.

[30] K.‐Y. Qian , Y. Song , X. Yan , L. Dong , J. Xue , Y. Xu , B. Wang , B. Cao , Q. Hou , W. Peng , J. Hu , K. Jiang , S. Chen , H. Wang , Y. Lu , Biomaterials 2020, 259, 120299. 32827797 10.1016/j.biomaterials.2020.120299

[31] R. J. R. Matos , C. I. P. Chaparro , J. C. Silva , M. A. Valente , J. P. Borges , P. I. P. Soares , Carbohydr. Polym. 2018, 198, 9. 30093046 10.1016/j.carbpol.2018.06.048

[32] J. Liao , H. Huang , Biomacromolecules 2020, 21, 2574. 32543834 10.1021/acs.biomac.0c00566

[33] S. Gil , J. M. Silva , J. F. Mano , ACS Biomater. Sci. Eng. 2015, 1, 1016. 33429532 10.1021/acsbiomaterials.5b00292

[34] Y.‐H. Chen , C.‐H. Cheng , W.‐J. Chang , Y.‐C. Lin , F.‐H. Lin , J.‐C. Lin , Mater. Sci. Eng. C 2016, 62, 338. 10.1016/j.msec.2016.01.070 26952432

[35] M. Criado , B. Sanz , G. F. Goya , C. Mijangos , R. Hernández , J. Mater. Chem. B 2017, 5, 8570. 32264525 10.1039/c7tb02361h

[36] A. Barra , Z. Alves , N. M. Ferreira , M. A. Martins , H. Oliveira , L. P. Ferreira , M. M. Cruz , M. D. D. Carvalho , S. M. Neumayer , B. J. Rodriguez , C. Nunes , P. Ferreira , J. Mater. Chem. B 2020, 8, 1256. 31960003 10.1039/c9tb02067e

[37] A. F. Alves , S. G. Mendo , L. P. Ferreira , M. H. Mendonça , P. Ferreira , M. Godinho , M. M. Cruz , M. D. Carvalho , J. Nanopart. Res. 2016, 18, 27.

[38] A. Chauhan , S. Midha , R. Kumar , R. Meena , P. Singh , S. K. Jha , B. K. Kuanr , Biomater. Sci. 2021, 9, 2972. 33635305 10.1039/d0bm01705a

[39] S. Ghosh , D. Carty , S. P. Clarke , S. A. Corr , R. Tekoriute , Y. K. Gun'ko , D. F. Brougham , Phys. Chem. Chem. Phys. 2010, 12, 14009. 20922236 10.1039/c0cp00989j

[40] R. Hergt , S. Dutz , J. Magn. Magn. Mater. 2007, 311, 187.

[41] A. Rath , S. Mathesan , P. Ghosh , Soft Matter 2016, 12, 9210. 27786328 10.1039/c6sm01994c

[42] A. Ramos‐Sebastian , S. J. Gwak , S. H. Kim , Adv. Sci. 2022, 9, 2103863. 10.1002/advs.202103863 PMC8895130 35060366

[43] Y. Kim , X. Zhao , Chem. Rev. 2022, 122, 5317. 35104403 10.1021/acs.chemrev.1c00481 PMC9211764

[44] C. Mira‐Cuenca , T. Meslier , S. Roig‐Sanchez , A. Laromaine , A. Roig , ACS Appl. Polym. Mater. 2021, 3, 4959.

[45] R. R. Besser , A. C. Bowles , A. Alassaf , D. Carbonero , I. Claure , E. Jones , J. Reda , L. Wubker , W. Batchelor , N. Ziebarth , R. Silvera , A. Khan , R. Maciel , M. Saporta , A. Agarwal , Biomater. Sci. 2020, 8, 591. 31859298 10.1039/c9bm01430f PMC7141910

[46] Y.‐J. Kim , M. Ebara , T. Aoyagi , Adv. Funct. Mater. 2013, 23, 5753.

[47] Z.‐X. Sun , F.‐W. Su , W. Forsling , P.‐O. Samskog , J. Colloid Interface Sci. 1998, 197, 151. 9466855 10.1006/jcis.1997.5239

[48] C. A. Schneider , W. S. Rasband , K. W. Eliceiri , Nat. Methods 2012, 9, 671. 22930834 10.1038/nmeth.2089 PMC5554542

[49] O. Kazakova , R. Puttock , C. Barton , H. Corte‐León , M. Jaafar , V. Neu , A. Asenjo , J. Appl. Phys. 2019, 125, 60901.

[50] P. Twentyman , M. Luscombe , Br. J. Cancer 1987, 56, 279. 3663476 10.1038/bjc.1987.190 PMC2002206

