# Supplementary material for: Magnetic Chitosan Bionanocomposite Films as a Versatile Platform for Biomedical Hyperthermia
Source: Adv Healthc Mater. 2023 Dec 13;13(11):2303861. doi: 10.1002/adhm.202303861 (PMC11468069; doi:10.1002/adhm.202303861)
Supplement: Supplementary file 1 — Supporting Information [file ADHM-13-2303861-s001.pdf]

ADVANCED HEALTHCARE MATERIALS

# Supporting Information

for *Adv. Healthcare Mater.*, DOI 10.1002/adhm.202303861

Magnetic Chitosan Bionanocomposite Films as a Versatile Platform for Biomedical Hyperthermia

*Ana Barra, Jacek K. Wychowaniec, Danielle Winning, Maria Margarida Cruz, Liliana P. Ferreira, Brian J. Rodriguez, Helena Oliveira, Eduardo Ruiz-Hitzky, Cláudia Nunes*, Dermot F. Brougham* and Paula Ferreira**

Supporting Information

**Magnetic chitosan bionanocomposite films as a versatile platform for biomedical hyperthermia**

*Ana Barra, Jacek K. Wychowaniec, Danielle Winning, Maria Margarida Cruz, Liliana P. Ferreira, Brian J. Rodriguez, Helena Oliveira, Eduardo Ruiz-Hitzky, Cláudia Nunes,* Dermot F. Brougham,* Paula Ferreira**

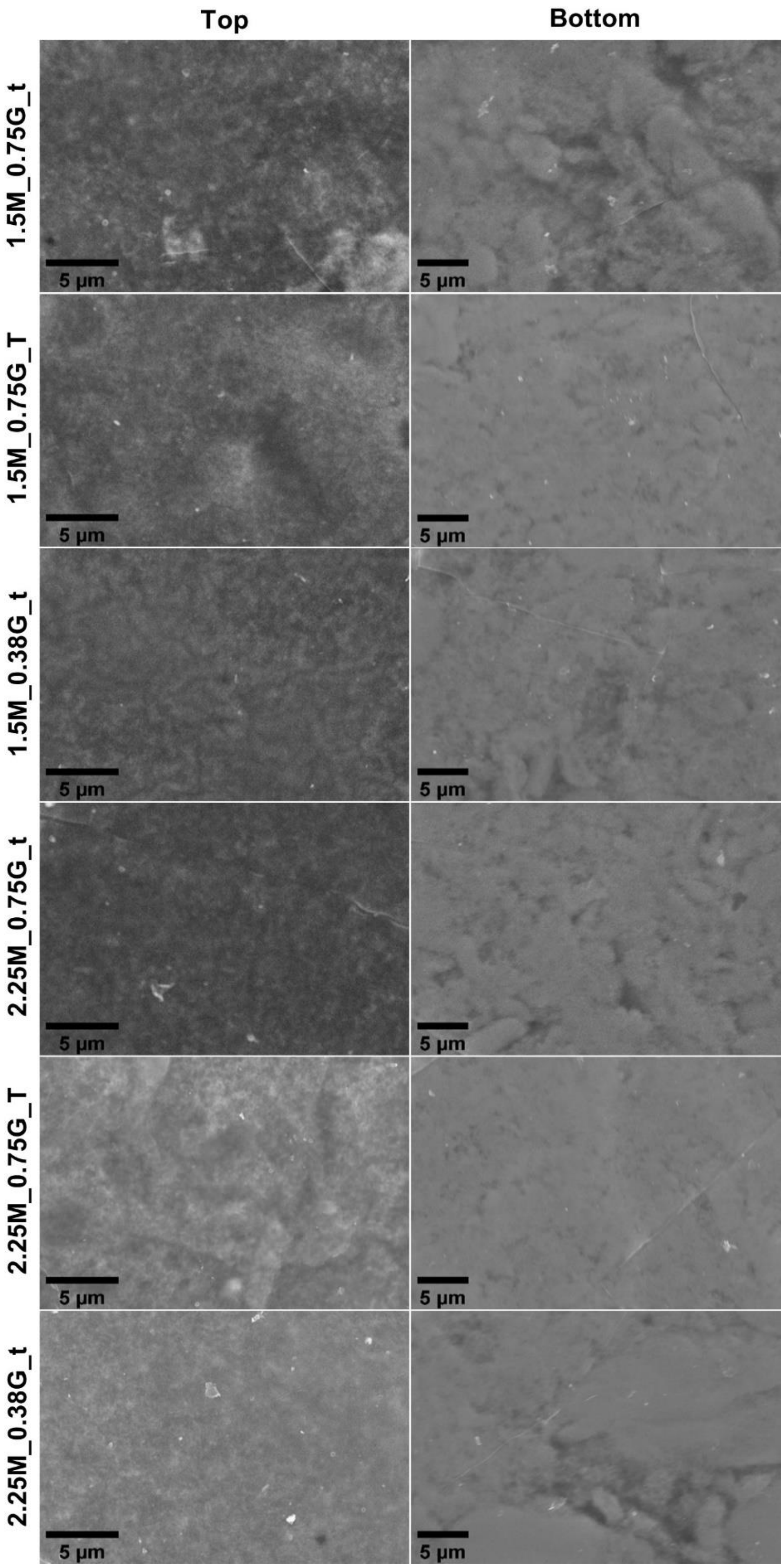


**Figure S1.** SEM micrographs of magnetic films. Left column, top. Right column, bottom.

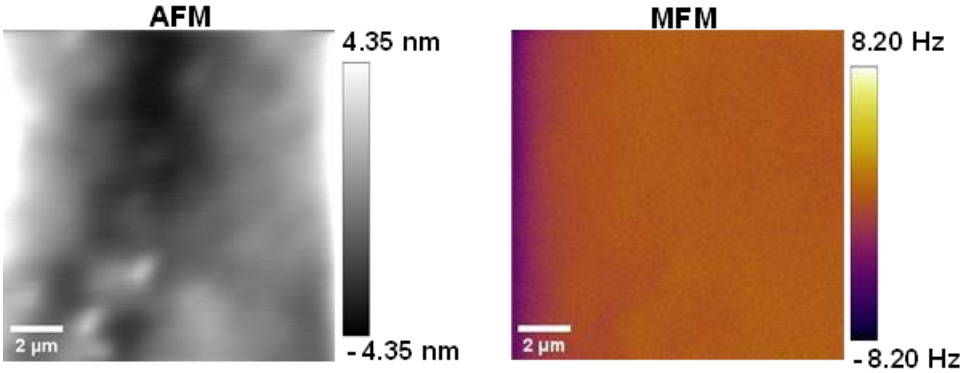


**Figure S2.** Topographic AFM image and the corresponding MFM frequency image of chitosan control film. The images were acquired in the same location with a lift height of 100 nm.

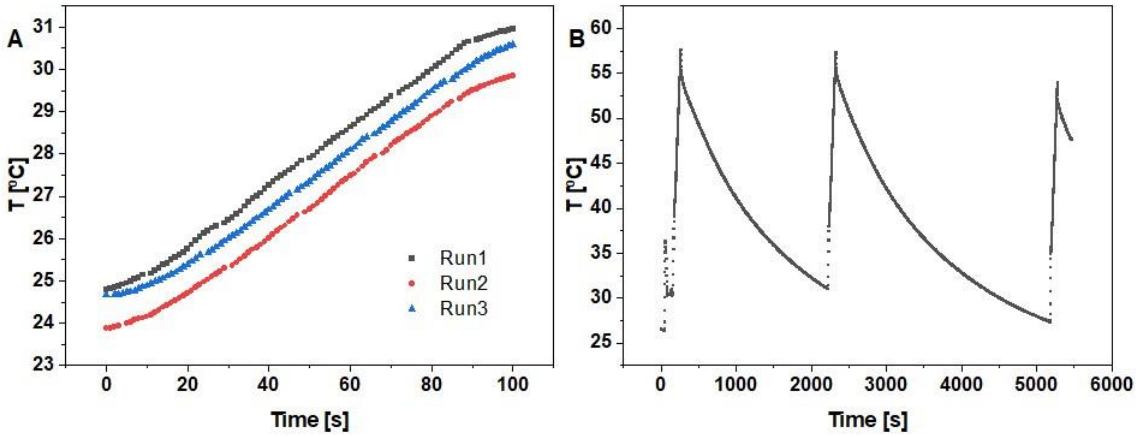


**Figure S3** Heating curves of 2.25M_0.75_T film in DMEM liquid media. (**A**) Heating curves of three different runs during 100 s (B) Heating curve with the AMF on and off. The measurements were performed using AMF at $\nu_{AC}$ 276 kHz, $H_{AC}$ 14.7 kA $m^{-1}$.

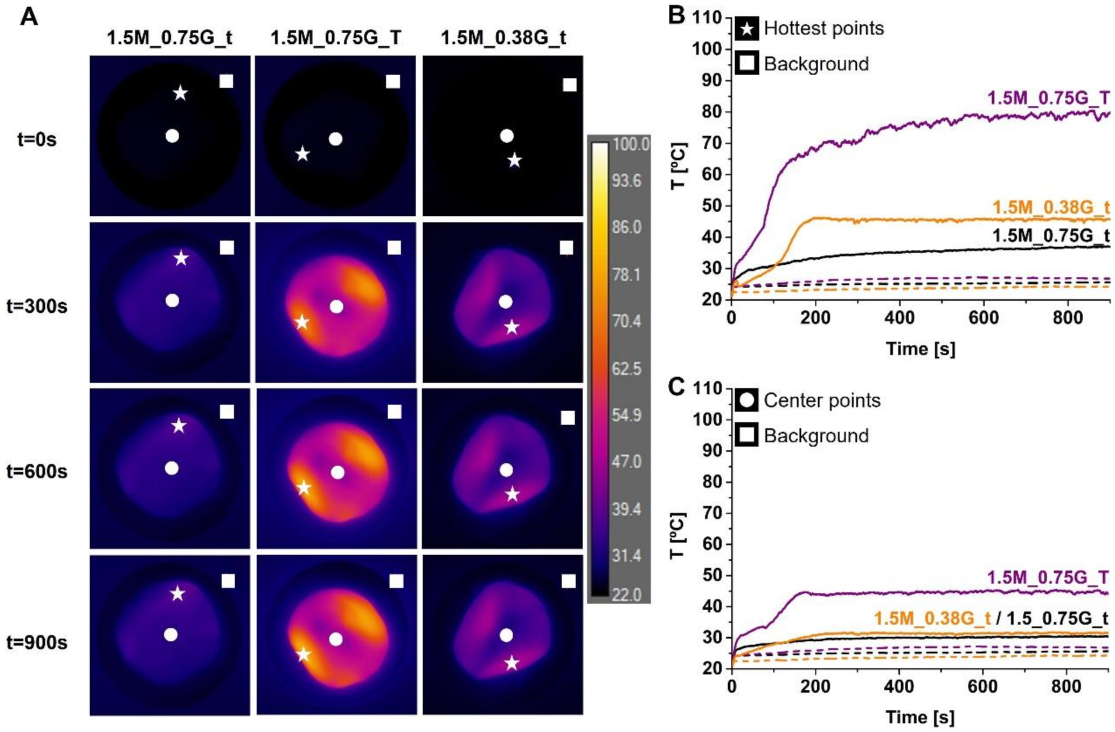


**Figure S4.** (**A**) LC-AMF thermal images of films containing 1.5 w/v% magnetite at t = 0, 300, 600 and 900 s, placed directly on PTFE tape on the LC-AMF setup. Measurements performed at a magnetic field strength of 16 mT and a frequency of 664.2 kHz. (**B**) Evolution of temperature in function of time in the hottest points (white stars, full line), and the corresponding background points (white squares, dashed lines). (**C**) Evolution of temperature in function of time in the centre points (white circles, full lines) and the corresponding background points (white squares, dashed lines).

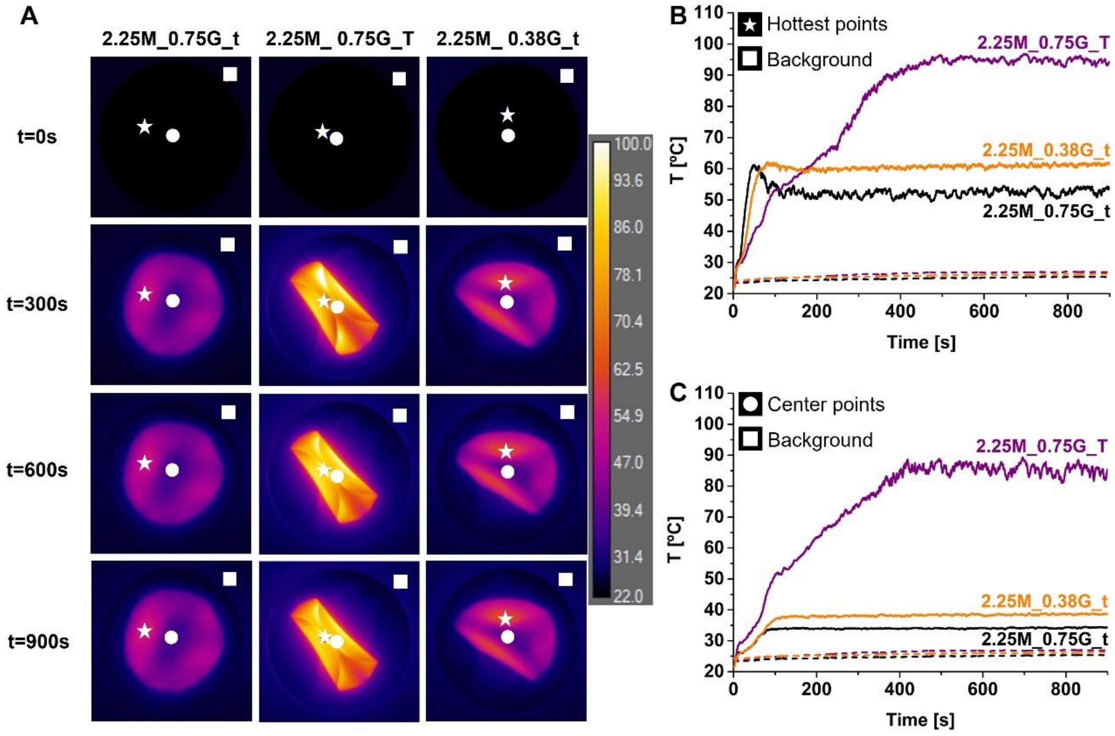


**Figure S5.** (**A**) LC-AMF thermal images of films containing 2.25 w/v% magnetite at t = 0, 300, 600 and 900 s, placed directly on PTFE tape on the LC-AMF setup. Measurements performed at a magnetic field strength of 16 mT and a frequency of 664.2 kHz. (**B**) Evolution of temperature in function of time in the hottest points (white stars, full line), and the corresponding background points (white squares, dashed lines). (**C**) Evolution of temperature in function of time in the centre points (white circles, full lines) and the corresponding background points (white squares, dashed lines).

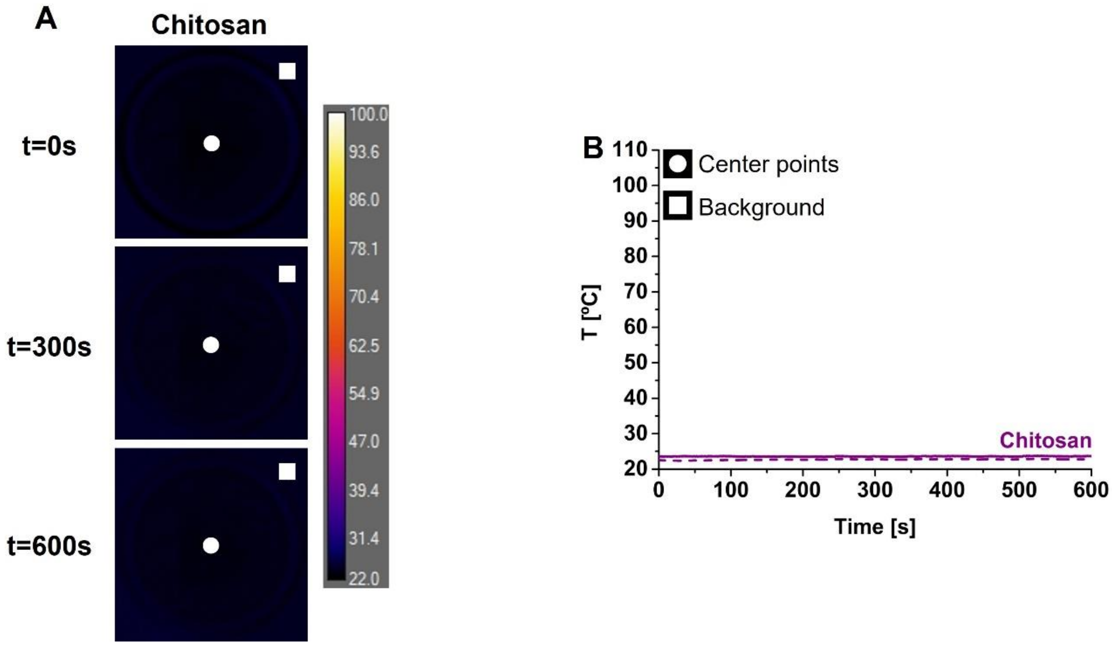


**Figure S6.** (**A**) LC-AMF thermal images of chitosan control film at t = 0, 300 and 600 s. Measurements performed at a magnetic field strength of 16 mT and a frequency of 664.2 kHz. (**B**) Evolution of temperature in function of time in the center point (white circle, full line) and the corresponding background point (white square, dashed line).

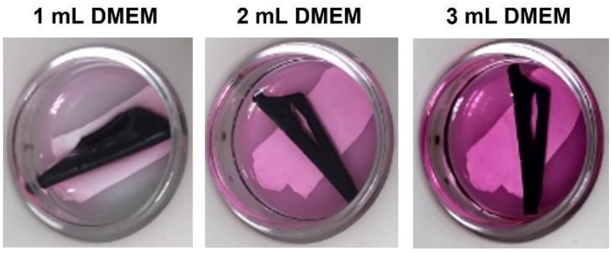


**Figure S7.** Digital images of the 2.25M_0.75G_T film covered with 1, 2 and 3 mL DMEM cell culture media.

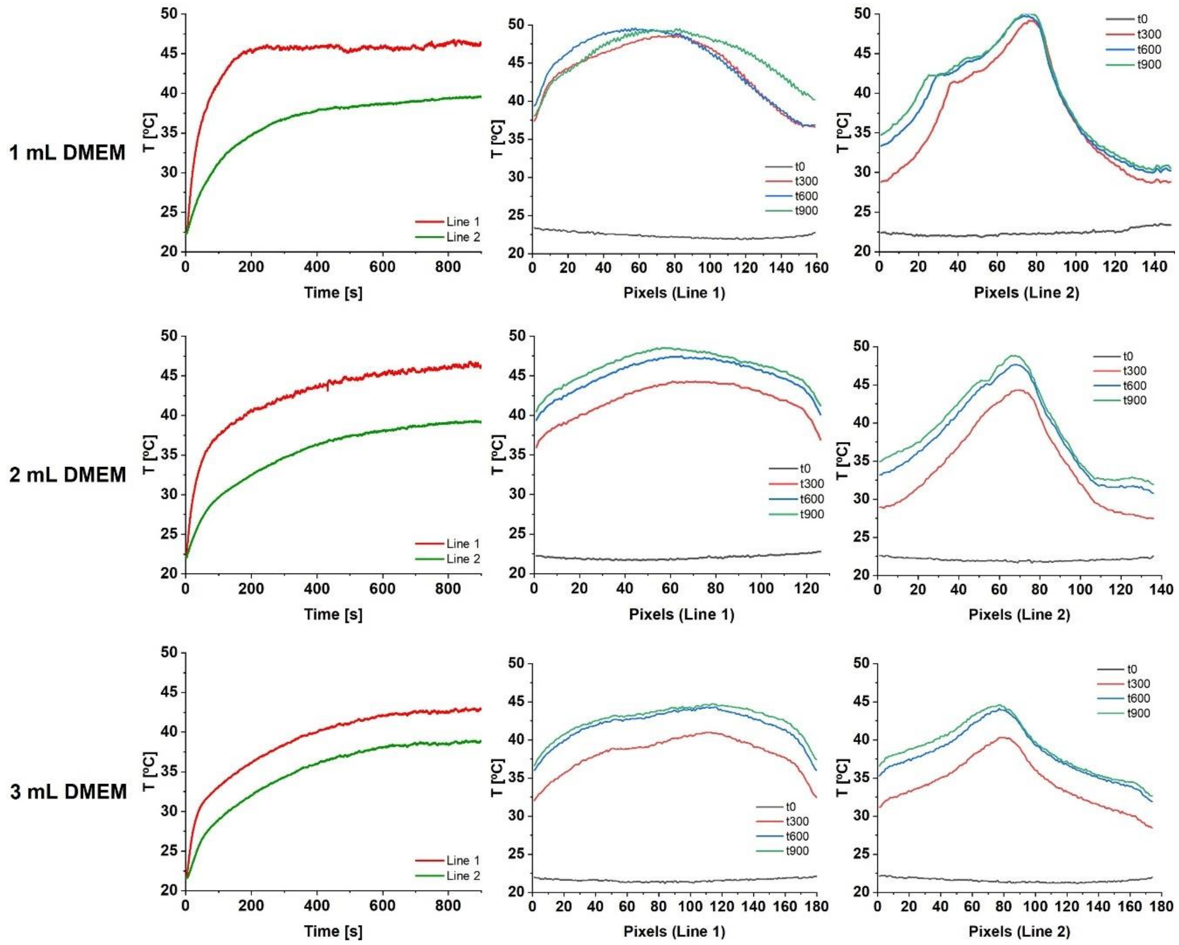


**Figure S8.** Evolution of temperature during LC-AMF of 2.25M_0.75G_T film at t = 0, 300, 600 and 900 s, placed on a glass Petri dish and covered with 1, 2 and 3 mL of DMEM cell culture medium. Line 1 and Line 2 are marked in the LC-AMF thermal images of Figure 6A.

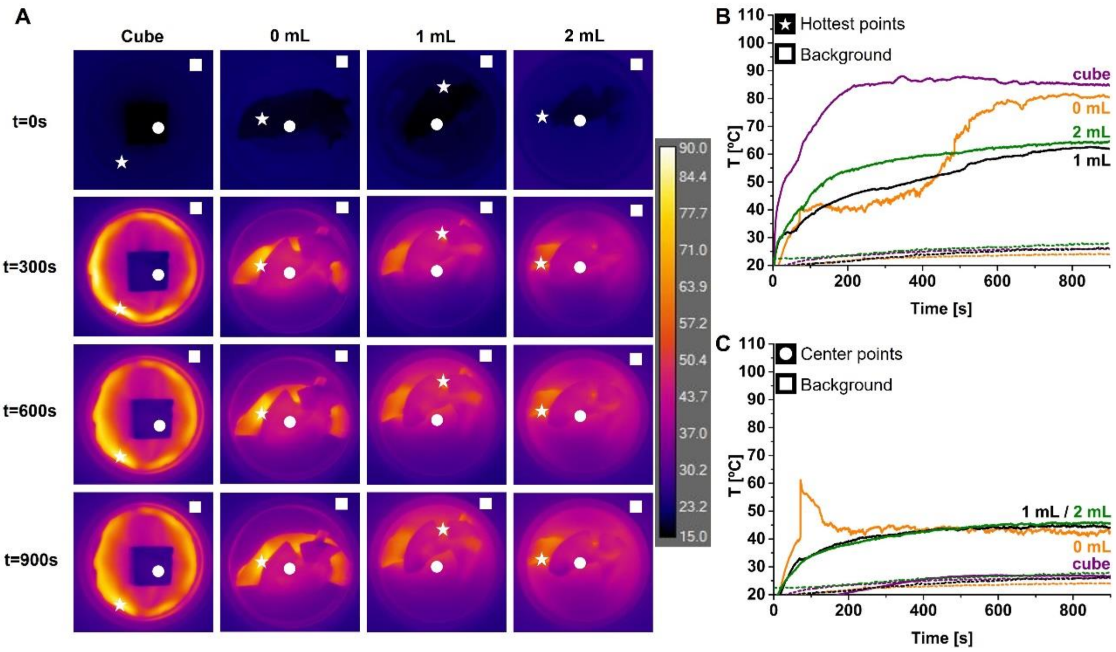


**Figure S9.** (**A**) LC-AMF thermal images of 2.25M_0.75G_T film with a 4 w/v% mTG crosslinked gelatin cuboid (0.6 mL) on top, and the 2.25M_0.75G_T film covered with a thin layer gel (0.6 mL) with 0, 1 and 2 mL DMEM cell culture medium on top. Measurements performed at a magnetic field strength of 16 mT and a frequency of 663 kHz. (**B**) Evolution of temperature in function of time in the hottest points (white stars, full line), and the corresponding background points (white squares, dashed lines). (**C**) Evolution of temperature in function of time in the centre points (white circles, full lines) and the corresponding background points (white squares, dashed lines).

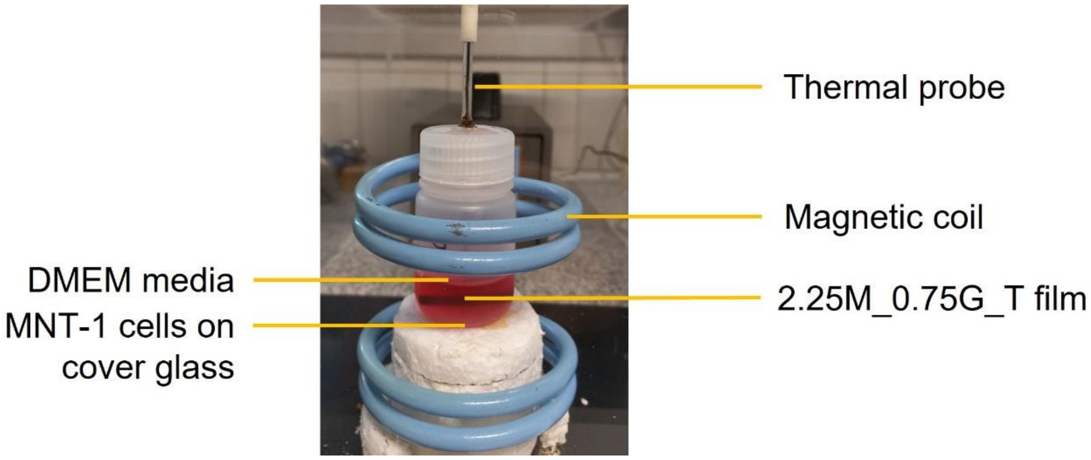


**Figure S10.** Scheme of magnetic hyperthermia setup.

**Supporting Movie 1 (SM1):** LC-AMF of 2.25M_0.75G_T film placed on a Petri dish on the LC-AMF setup.

https://www.dropbox.com/s/l319lysn2oukf89/SM1.wmv?dl=0

**Supporting Movie 2 (SM2):** LC-AMF of 2.25M_0.75G_T film placed directly on PTFE tape on the LC-AMF setup.

https://www.dropbox.com/s/sqg7jt4j0p2ct09/SM2.wmv?dl=0

**Supporting Movie 3 (SM3):** Demonstration of 2.25M_0.75G_T film folding upon water/ethanol solvent exchange.

https://www.dropbox.com/s/ynb8qhjo0kiaqve/SM3.mp4?dl=0

**Supporting Movie 4 (SM4):** LC-AMF of 2.25M_0.75G_T with a cuboid-shaped 10 w/v% mTG-Gel on top.

https://www.dropbox.com/s/ki7cm6sasqvs8vm/SM4.wmv?dl=0

**Supporting Movie 5 (SM5):** LC-AMF of 2.25M_0.75G_T covered with parafilm, a 0.6mL layer of 10 w/v% gelatine and 3 mL DMEM.

https://www.dropbox.com/s/2kx42vfcl6olv8r/SM5.wmv?dl=0
